# CARE TO PLAN: TAILORING RESOURCES FOR DEMENTIA CAREGIVERS ACROSS A HEALTH SYSTEM

**DOI:** 10.1093/geroni/igac059.1771

**Published:** 2022-12-20

**Authors:** Ashley Millenbah, Katie Louwagie, Zachary Baker, Christine Jensen, Joseph Gaugler

**Affiliations:** University of Minnesota, Minneapolis, Minnesota, United States; University of Minnesota, Minneapolis, Minnesota, United States; Arizona State University, Tempe, Arizona, United States; Riverside Center for Excellence in Aging and Lifelong Health, Williamsburg, Virginia, United States; University of Minnesota, Minneapolis, Minnesota, United States

## Abstract

Each day, millions of persons with Alzheimer’s disease or related dementias (ADRD) rely on family members for necessary care and support. While there are many well-documented interventions to support ADRD caregivers, they lack individualization for specific caregiver needs and contexts. Care to Plan (CtP) was designed to address this gap in tailoring recommendations to support caregivers. After a Phase I pilot, CtP was refined and implemented within a moderately sized health system in the eastern US. A mixed-methods randomized controlled trial aimed to provide insights into the efficacy, acceptability, and utility of CtP. Twenty-two caregivers were randomly assigned to the treatment group (to review the CtP tool with a senior care navigator) and 21 were assigned to receive usual care. After completion of usual care procedures, 7 participants joined a waitlist control group, following treatment procedures. Three- and six-month outcomes included caregiver self-efficacy and distress, caregiver use of recommended support, and service utilization by the person with memory loss. Most caregivers were white (81%), female (74%), and cared for a parent (56%) or spouse (35%). The majority of caregivers agreed that the tool was helpful (70%) and would recommend it to other caregivers (83%). While 48% of caregivers felt that resources provided by the tool were not new to them, the checklist scores and interviews indicate that the senior care navigators were valuable in discussing the recommendations. Phase II findings will guide future evaluation and dissemination, and inform future models of CtP for health system usage.

